# Supplementary material for: Genome-Scale Modeling Specifies the Metabolic Capabilities of Rhizophagus irregularis
Source: mSystems. 2022 Jan 25;7(1):e01216-21. doi: 10.1128/msystems.01216-21 (PMC8793856; doi:10.1128/msystems.01216-21)
Supplement: TEXT S1 [file msystems.01216-21-t0001.docx]

**iRi1574 MEMOTE score.** The stoichiometric consistency, mass- and charge-balancing, metabolite connectivity, as well as thermodynamic consistency scored with 25%, 85.8%, 95.4%, 99.9%, and 98.2%, demonstrating that the MEMOTE score is mainly influenced by cross-referencing and feature annotation. Without cross-references, the score dropped to 40%, due to the large weight on reaction-, metabolite-, and gene annotation. The thermodynamic consistency is affected by the presence of stoichiometric balanced cycles (SBCs). MEMOTE reported that 13 reactions appear in SBCs, and thus, carry unbounded flux; however, these reactions are comprised of 11 transport and sink reactions and two enzymatic reactions (Table S1M). These included the diacyl-o-acyltransferase and the triacylglycerol hydrolase reactions that catalyse the conversion of diacylglycerol (DAG) species to triacylglycerol (TAG) species and vice versa. The flux through the triacylglycerol acylhydrolase reaction can be maximized to 999.8 mmol/gDW/h but is not required for growth of the model and carries no flux in FBA, indicating that it likely not involved in a SBC relevant to the study of growth.

**MOMENT, GECKO, and eMOMENT.** Both MOMENT(1) and GECKO(2) are approaches that enable the integration of enzyme abundance constraints into metabolic models. Both methods impose upper bounds on reaction fluxes based on either the distribution of total protein among enzymes (MOMENT, enzyme pool in GECKO) and/or allow the direct integration of enzyme abundances (GECKO). In contrast to MOMENT, GECKO augments the stoichiometric matrix with pseudo and so-called “arm” reactions and pseudometabolites (corresponding to enzymes) to implicitly accounts for enzyme promiscuity as well as for isoenzymes. However, enzyme promiscuity is not considered in MOMENT.

For our analysis, we chose to apply the less-constrained MOMENT approach, which only imposes upper bounds based on enzyme abundance, and does not necessitate the addition of “arm reactions”. However, to deal with enzyme promiscuity, we added additional variables to the constraint-based problem, which represent the specific amount of every enzyme available for each of its catalysed reactions ($E_{i}^{r}$). Thus, the sum of all $E_{i}^{r}$ associated to the protein product of gene $k$ must equal the total amount of the respective protein abundance in the simulation:

$$\sum_{k=1}^{|Genes|} E_{i\in GPR_{k}}^{r}=E_{k}^{g}$$

Notably, this converges to the same constraints on enzyme promiscuity that are imposed by GECKO. However, we did not split reactions with isozymes to introduce “arm reactions”, so we termed the implemented approach *eMOMENT* for distinction. Therefore, eMOMENT, like GECKO, accounts from both isoenzymes and promiscuous enzymes, but, unlike GECKO, does not require network structure encoding of the constraints. In addition, as pointed out in the Methods, eMOMENT includes binary variables to model when a protein is considered expressed.

**Experimental procedures (carried over from previously published studies, not from this study).** Our simulation with different carbon sources and concentrations was conducted using data from an experiment conducted by Hildebrandt and colleagues (3). The authors used the AMF fungus *Glomus intraradices* Sy 167 for this experiment; *Glomus intraradices* Sy 167 was grown on a modified M-Medium (4) and 0.4% (w/v) gellan gum (GelGro, ICN, D-Eschwege, Germany) supplied with different concentrations (10 mM, 100 mM, and 1 M) of five oligosaccharides (i.e. raffinose, trehalose, melibiose, glucose, and fructose). The experiment was started by adding about 1000 spores that were obtained after growing the fungus with Ri T-DNA transformed carrot roots. The fungal material was harvested after 2.5 month by solubilization of the GelGro with citrate buffer (pH 6.0) at 30 °C and grinding in liquid nitrogen.

The protein content was determined by adding 40 µl of extraction buffer to material harvested from three plates. This buffer was composed of 10 mM Tris-HCL (pH 8.0), 1 mM EDTA, 2% polyvinylpolypyrrolidone, 0.5% leupeptin, and 0.5% phenylmethylsulfonylfluoride (w/v). The lysate was then centrifuged at 4 °C for 30 min, and the protein content was finally determined by the Lowry method (5).

For our analysis, we used the measurements of total hyphal weight and protein content given in Table 2 of the original publication (3).

For the integration of expression data into the iRi1572 model, we re-analyzed data published by Zeng et al. (2018) (6) (NCBI Gene Expression Omnibus accession GSE99655). To generate these data, the authors used laser microdissection to obtain fungal material from seven-week-old Medicago roots infected with *Rhizophagus irregularis* DAOM197198. As the precise preparation details can be found in the Material and Methods section of the original study, in the following, we only refer to the major steps undertaken in the experiment.

The root material was first cleaned and fixed. The fixed roots were then dehydrated and embedded in Steedman wax. The roots were cut using a microtome, stretched on a PEN-membrane slide, and the Steedman wax was removed. The fungal material (i.e. arbuscule cells, intraradical mycelium cells) was collected into an Eppendorf tube. Like this, about 2000 cells were collected for each arbuscule replicate and about 10000 cells were collected for each of the intraradical mycelium replicates

The RNA was subsequently isolated from slides that showed sufficient overall RNA quality. After this step, 2 µl RNA of each sample was transcribed into to cDNA, pre-amplified, and again quality-checked by qPCR using mycorrhiza-induced marker genes. The best-performing replicates for each cell type were then amplified using 10-15 cycles according to the respective RNA content determined earlier.

The sequencing libraries were made using ThruPLEX DNA-seq Kit and sequenced on an Illumina HiSeq 2500 platform (paired-end, 125 bp x 2). The generated reads were mapped to the *R. irregularis* genome published by Lin and co-workers (7).

For our analysis, we re-quantified the reads as described in the Material and Methods section of our manuscript, using the same annotation that we used to reconstruct the metabolic model (8, 9).

**References**

1. Adadi R, Volkmer B, Milo R, Heinemann M, Shlomi T. 2012. Prediction of microbial growth rate versus biomass yield by a metabolic network with kinetic parameters. PLoS Comput Biol 8:e1002575.

2. Sánchez BJ, Zhang C, Nilsson A, Lahtvee P, Kerkhoven EJ, Nielsen J. 2017. Improving the phenotype predictions of a yeast genome‐scale metabolic model by incorporating enzymatic constraints. Mol Syst Biol 13:935.

3. Hildebrandt U, Ouziad F, Marner F-J, Bothe H. 2006. The bacterium *Paenibacillus validus* stimulates growth of the arbuscular mycorrhizal fungus *Glomus intraradices* up to the formation of fertile spores. FEMS Microbiol Lett 254:258–267.

4. Bécard G, Fortin JA. 1988. Early events of vesicular–arbuscular mycorrhiza formation on Ri T‐DNA transformed roots. New Phytol 108:211–218.

5. Lowry OH, Rosebrough NJ, Farr AL, Randall RJ. 1951. Protein measurement with the Folin phenol reagent. J Biol Chem 193:265–275.

6. Zeng T, Holmer R, Hontelez J, Lintel‐Hekkert B, Marufu L, Zeeuw T, Wu F, Schijlen E, Bisseling T, Limpens E. 2018. Host‐ and stage‐dependent secretome of the arbuscular mycorrhizal fungus *Rhizophagus irregularis*. Plant J 94:411–425.

7. Lin K, Limpens E, Zhang Z, Ivanov S, Saunders DGO, Mu D, Pang E, Cao H, Cha H, Lin T, Zhou Q, Shang Y, Li Y, Sharma T, van Velzen R, de Ruijter N, Aanen DK, Win J, Kamoun S, Bisseling T, Geurts R, Huang S. 2014. Single Nucleus Genome Sequencing Reveals High Similarity among Nuclei of an Endomycorrhizal Fungus. PLoS Genet 10.

8. Chen ECH, Morin E, Beaudet D, Noel J, Yildirir G, Ndikumana S, Charron P, St-Onge C, Giorgi J, Krüger M, Marton T, Ropars J, Grigoriev I V., Hainaut M, Henrissat B, Roux C, Martin F, Corradi N. 2018. High intraspecific genome diversity in the model arbuscular mycorrhizal symbiont *Rhizophagus irregularis*. New Phytol 220:1161–1171.

9. Tisserant E, Malbreil M, Kuo A, Kohler A, Symeonidi A, Balestrini R, Charron P, Duensing N, Frei dit Frey N, Gianinazzi-Pearson V, Gilbert LB, Handa Y, Herr JR, Hijri M, Koul R, Kawaguchi M, Krajinski F, Lammers PJ, Masclaux FG, Murat C, Morin E, Ndikumana S, Pagni M, Petitpierre D, Requena N, Rosikiewicz P, Riley R, Saito K, San Clemente H, Shapiro H, van Tuinen D, Becard G, Bonfante P, Paszkowski U, Shachar-Hill YY, Tuskan GA, Young JPW, Sanders IR, Henrissat B, Rensing SA, Grigoriev I V., Corradi N, Roux C, Martin F. 2013. Genome of an arbuscular mycorrhizal fungus provides insight into the oldest plant symbiosis. Proc Natl Acad Sci 110:20117–20122.
