# Supplementary material for: Genome-Scale Modeling Specifies the Metabolic Capabilities of Rhizophagus irregularis
Source: mSystems. 2022 Jan 25;7(1):e01216-21. doi: 10.1128/msystems.01216-21 (PMC8793856; doi:10.1128/msystems.01216-21)
Supplement: TEXT S2 [file msystems.01216-21-t0002.docx]

**Supplementary Methods**

**Adjustment of coefficients in SLIMEr reactions**

The SLIMEr approach (1) was used to increase the accuracy of the lipid metabolism in the iRi1574 model. The publicly available lipid abundance data for lipid species (2) and fatty acids (3) were used in the SLIMEr reactions that pull lipid backbones and chains, respectively. However, using these data directly as coefficients did not yield a feasible optimization problem in FBA. Thus, we aimed at finding minimal adjustments to the coefficients $s_{b}$ and $s_{c}$ for the backbone ($B$) and chain ($C$) pseudometabolites in the final SLIMEr reaction that produces the lipid entity, which in turn fuels the biomass reaction:

$$s_{b}B+s_{c}C\to L.$$

Therefore, we devised a quadratically-constraint quadratic optimization problem (QCQP), which minimizes the second norm of adjustment variables $\varepsilon_{b}$ and $\varepsilon_{c}$, which are added to the respective original coefficients (i.e. -1). The stoichiometric matrix was hence altered by transforming the steady state constraints for $B$ and $C$ into quadratic constraints where the original stoichiometric coefficients are replaced:

$$\left( s_{b}+\varepsilon_{b} \right)v_{S}+\left( s_{c}+\varepsilon_{b} \right)v_{S}+s_{L}v_{S}=0,$$

where $v_{S}$ represents the flux variable for the reaction shown above and $s_{L}$ is the stoichiometric coefficient of the resulting lipid pseudometabolite (i.e. 1). The following optimization problem, the altered stoichiometric matrix is represented by $S^{*}$:

$$\min\left\| \varepsilon_{b}+\varepsilon_{c} \right\|$$

s.t.

$$S^{*}v=0$$

$$v_{i}^{min}\leq v_{i}\leq v_{i}^{max}, \forall i\in R.$$

This QCQP was solved using the Gurobi solver (version 9.1.1) with adjusted parameters as shown below:

NonConvex: 2

NumericFocus: 3

MIPFocus: 3

FeasibilityTol: 1e-9.

The adjusted coefficients were $s_{b}^{*}=-1.1533$ and $s_{c}^{*}=-0.2294$.

**Comparison of fungal models**

Genome-scale metabolic models were obtained for 11 fungal species (Suppl. Tab 2) and imported into Matlab (4) via functions of the COBRA (5) and RAVEN 2.0 (6) toolboxes depending on the their format. Two of the models were discarded because files could not be read or no E.C. numbers were available. The reactions of the remaining nine models were mapped to the subsystems as defined in the YeastGEM v8.3.4 model (7) by matching associated E.C. numbers. As a result, subsystems could be assigned to 40% to 55% of reactions in the respective models. The overlap of E.C. numbers per subsystem with the iRi1574 model was quantified by calculating the Jaccard Index.

For functional characterization, the minimal and maximal sums of fluxes as well as the sum of fluxes resulting from parsimonious FBA through each of the subsystems of interest were determined. Altogether, we chose eight subsystems for this comparison: ‘Biosynthesis of amino acids’, ‘Carbon metabolism’, ‘Fatty acid metabolism’, ‘Purine metabolism’, ‘Pyrimidine metabolism’, ‘Glycolysis’, ‘Citrate cycle (TCA cycle)’, and ‘Arginine biosynthesis’. The maximal and minimal sums of fluxes were determined by running the following LP:

$$\text{min/}\text{max}\sum_{i\in R_{subsystem}} v_{i}$$

s.t.

$$\boldsymbol{Sv=0}$$

$$v_{bio}\geq0.9\cdot v_{bio}^{opt}$$

$$v_{i}^{min}\leq v_{i}\leq v_{i}^{max}, \forall i\in R,$$

where $R$ is the set of all reactions, $S$ is the stoichiometric matric, and $v_{bio}^{opt}$ is the optimal biomass as obtained from FBA. For parsimonious FBA, the sum of fluxes was minimized while guaranteeing for optimal growth:

$$\min\sum_{i\in R} \left| v_{i} \right|$$

s.t.

$$\boldsymbol{Sv=0}$$

$$v_{bio}=v_{bio}^{max}$$

$$v_{i}^{min}\leq v_{i}\leq v_{i}^{max}, \forall i\in R.$$

Both LPs were run using the COBRA toolbox function *optimizeCbModel* with appropriate parameters.

**Prediction of growth upon myristate addition using the enzyme-constraint model**

For this analysis, we first determined the minimum uptake of palmitate that guarantees optimal growth using the eMOMENT approach. Subsequently, we determined the minimal uptake fluxes of additional carbon sources at optimal growth by minimizing the sum of their fluxes. We then set the upper bound for palmitate uptake to 10% of the minimum flux determined before. The molecular crowding constraint from the eMOMENT MILP was removed and replaced by two separate constraints for the peroxisomal pool of proteins and all remaining proteins for a series of 1000 equally spaced values $\alpha$ between zero and one. For each of these ratios, we predicted a reference growth rate without the addition of myristate to the medium. The growth rate predicted upon allowing for unlimited uptake of myristate was then divided by the reference value to obtain the ratio $Z^{\alpha}$at a given $\alpha$.

**Calculation of specific growth rates**

Specific growth rates $\mu$ were calculated from initial spore dry weight $w_{0}$, growth duration $t$, and final hyphae dry weight $w_{t}$, assuming exponential growth:

$$w_{t}=w_{0}\cdot e^{\mu t}.$$

By re-arranging for $\mu$, we obtain:

$$\mu=\ln\left( \frac{w_{t}}{w_{0}} \right)\cdot t^{-1}.$$

The initial dry weight of the spores was calculated by the number of parent spores $n_{p}$, multiplied with the dry weight per parent spore $w_{p}$:

$$w_{0}=n_{p}\cdot w_{p}.$$

The values $w_{t}$, $t$, and $n_{p}$ were taken from the experimental setup that was used in (8) (*G. intraradices* Sy 167, see Table 2 and Method section in (8)). The weight per parent spore ($w_{p}=0.3 \mu g$) was obtained from the work of Sugiura and colleagues (9) (*R. irregularis* DAOM197198, see Fig. S1A in (9)).

**References**

1. Sánchez BJ, Li F, Kerkhoven EJ, Nielsen J. 2019. SLIMEr: Probing flexibility of lipid metabolism in yeast with an improved constraint-based modeling framework. BMC Syst Biol 13:1–9.

2. Wewer V, Brands M, Dörmann P. 2014. Fatty acid synthesis and lipid metabolism in the obligate biotrophic fungus *Rhizophagus irregularis* during mycorrhization of *Lotus japonicus*. Plant J 79:398–412.

3. Olsson PA, Johansen A. 2000. Lipid and fatty acid composition of hyphae and spores of arbuscular mycorrhizal fungi at different growth stages. Mycol Res 104:429–434.

4. 2017. MATLAB version 9.9.0.1524771 (R2020b) Update 2. The Mathworks, Inc., Natick, Massachusetts.

5. Heirendt L, Arreckx S, Pfau T, Mendoza SN, Richelle A, Heinken A, Haraldsdóttir HS, Wachowiak J, Keating SM, Vlasov V, Magnusdóttir S, Ng CY, Preciat G, Žagare A, Chan SHJ, Aurich MK, Clancy CM, Modamio J, Sauls JT, Noronha A, Bordbar A, Cousins B, El Assal DC, Valcarcel L V., Apaolaza I, Ghaderi S, Ahookhosh M, Ben Guebila M, Kostromins A, Sompairac N, Le HM, Ma D, Sun Y, Wang L, Yurkovich JT, Oliveira MAP, Vuong PT, El Assal LP, Kuperstein I, Zinovyev A, Hinton HS, Bryant WA, Aragón Artacho FJ, Planes FJ, Stalidzans E, Maass A, Vempala S, Hucka M, Saunders MA, Maranas CD, Lewis NE, Sauter T, Palsson BØ, Thiele I, Fleming RMT. 2019. Creation and analysis of biochemical constraint-based models using the COBRA Toolbox v.3.0. Nat Protoc 14:639–702.

6. Wang H, Marcišauskas S, Sánchez BJ, Domenzain I, Hermansson D, Agren R, Nielsen J, Kerkhoven EJ. 2018. RAVEN 2.0: A versatile toolbox for metabolic network reconstruction and a case study on *Streptomyces coelicolor*. PLOS Comput Biol 14:e1006541.

7. Lu H, Li F, Sánchez BJ, Zhu Z, Li G, Domenzain I, Marcišauskas S, Anton PM, Lappa D, Lieven C, Beber ME, Sonnenschein N, Kerkhoven EJ, Nielsen J. 2019. A consensus *S. cerevisiae* metabolic model Yeast8 and its ecosystem for comprehensively probing cellular metabolism. Nat Commun 10:3586.

8. Hildebrandt U, Ouziad F, Marner F-J, Bothe H. 2006. The bacterium *Paenibacillus validus* stimulates growth of the arbuscular mycorrhizal fungus *Glomus intraradices* up to the formation of fertile spores. FEMS Microbiol Lett 254:258–267.

9. Sugiura Y, Akiyama R, Tanaka S, Yano K, Kameoka H, Marui S, Saito M, Kawaguchi M, Akiyama K, Saito K. 2020. Myristate can be used as a carbon and energy source for the asymbiotic growth of arbuscular mycorrhizal fungi. Proc Natl Acad Sci 117:202006948.
